# Correlation Study of Biological Activity with Quercetin and Phenolics Content in Onion Extracts

**DOI:** 10.3390/molecules27238164

**Published:** 2022-11-23

**Authors:** Małgorzata Olszowy-Tomczyk, Sylwia Garbaczewska, Dorota Wianowska

**Affiliations:** 1 Department of Chromatography, Institute of Chemical Sciences, Faculty of Chemistry, Maria Curie-Skłodowska University in Lublin, Pl. Maria Curie-Skłodowska 3, 20-031 Lublin, Poland; 2 Department of Synthesis and Technology, Institute of Industrial Organic Chemistry, 6 Annopol St., 03-236 Warsaw, Poland

**Keywords:** *Allium cepa*, onion extracts activity, antioxidant activity, antifungal activity, quercetin, correlation

## Abstract

In this study it was shown that the fungistatic and antioxidant activities of onion extracts are related to the type of liquid used as the extractant and the technique of its preparation. A change in the antioxidant properties of white and red onion extracts was demonstrated with the change of the temperature of the pressurized hot water extraction process, which can be easily related to the changes accompanying the process of thermal processing of vegetables and fruits during cooking. Owing to the experimental and mathematical approaches concerning both the main and characteristic components of onions, i.e., quercetin and phenols, respectively, with the biological activity of the extracts, it was possible to demonstrate the significant share of these compounds in the antifungal and antioxidant properties of the extracts. Considering that the research was carried out, inter alia, on onion husks, demonstrating a very high potential of biological properties of this waste material from agricultural production, the research results presented in the paper should encourage the popularization of the use of this so far underestimated raw material for the production of various functional materials.

## 1. Introduction

The growing proportion of elderly people in many world populations and the emergence of various age-related metabolic disorders increase consumer health awareness and interest in a healthy diet rich in compounds with significant health benefits [1]. One such compound is quercetin, a polyphenolic antioxidant commonly consumed in many fruits and vegetables. The compound has the potential to protect against cancer and heart disease, the two most common causes of death in industrialized countries [2]. Quercetin is also attributed to anti-inflammatory, antibacterial, anti-obesity, antihypercholesterolemic and even anti-HIV properties [3,4]. As such, it is not surprising that this compound is of interest for plant breeders, food technologists and nutritionists alike. It is used to evaluate plant breeding lines for genetic selection and to monitor the quality of plant products during their storage and processing.

The interest in the health-promoting properties of quercetin results in a significant increase in the demand for this compound. Its new sources are being searched for, paying more and more attention to materials that so far have been neglected, such as post-production agricultural waste. More efficient and environmentally friendly extraction techniques are also being sought. Consequently, now the literature offers a number of different approaches to quercetin extraction from plants, including the edible and inedible parts of different varieties of onion (*Allium cepa* L.), as one of the most consumed vegetables in the world that is additionally rich in quercetin. Most often, these are modern approaches classified as miniaturized or assisted extraction techniques. The former include simple techniques useful on a laboratory scale for assessment of the actual quercetin content in the plant material [5,6]. The latter, in turn, are advanced approaches, compliant with the principles of green chemistry and useful for the isolation of quercetin on an industrial scale [7,8,9].

The literature reports also the papers combining the use of pro-ecological techniques of quercetin isolation with superheated water with the possibility of a mathematical design of extraction conditions for the optimal yield of this compound, showing a wide range of possible biological activities, including antioxidant and antifungal ones of quercetin-rich isolates obtained from the onion waste materials [10]. This research trend is in line with the “zero-waste” philosophy, the results of which can be useful in many different industries. Therefore, this study is a continuation of the previous investigations. Its aim is to investigate the correlation between the content of quercetin as the main component of extracts and the biological activity of onion extracts, as well as the sum of polyphenols and the activity of these extracts. Apart from the pressurized hot water extraction (PHWE) technique, the conventional extraction in the Soxhlet apparatus and maceration were applied in the research. In both, methanol, acetone, ethyl acetate, and chloroform were used as the extraction solvents. The antioxidant activity was assessed by the ABTS, DPPH and beta-carotene methods. The antifungal activity was tested against various plant and bee pathogenic fungi. The Pearson’s and Spearman’s coefficients were used to assess the correlation.

## 2. Results and Discussion

As it is commonly known, the biological activity of natural extracts is the result of the synergistic and antagonistic activity of the compounds present in the extracts [11]. Taking into account the very complex and complicated nature of the extracts, the research most often compares the activity of the extract with that of the main compounds characteristic of a given plant material. In the case of onion, these are phenolic compounds, especially quercetin. Therefore, this study was assumed to start with determining the content of these substances in the extracts obtained from onion scales, using various techniques and solvents, including water, i.e., liquids classified as GRAS class reagents (Generally Recognized As Safe). The extracts were obtained by Pressurized Hot Water Extraction (PHWE) process, maceration (Mac) and extraction in the Soxhlet apparatus (Sox) with methanol (MeOH), acetone (Ac), ethyl acetate (EtOAc), and chloroform (CHCl3), i.e., with solvents of various polarities (selectivities). Table 1 summarizes the amount of quercetin determined in the tested extracts using the HPLC technique and the total content of phenolic compounds determined spectrophotometrically.

The values listed in Table 1 prove that the content of the tested compounds in the onion extracts is diversified and largely dependent on the applied extractant type and the extraction temperature. The greatest amount of quercetin is found in the EtOAC and MeOH extracts obtained during maceration. The use of extraction in the Soxhlet apparatus reduces the quercetin yield, which confirms the instability of the compound to long-term exposure to high temperature, as known from the literature [5,6,7,8,9,10]. The smallest amounts of quercetin are present in the chloroform extracts.

**Figure 1:**
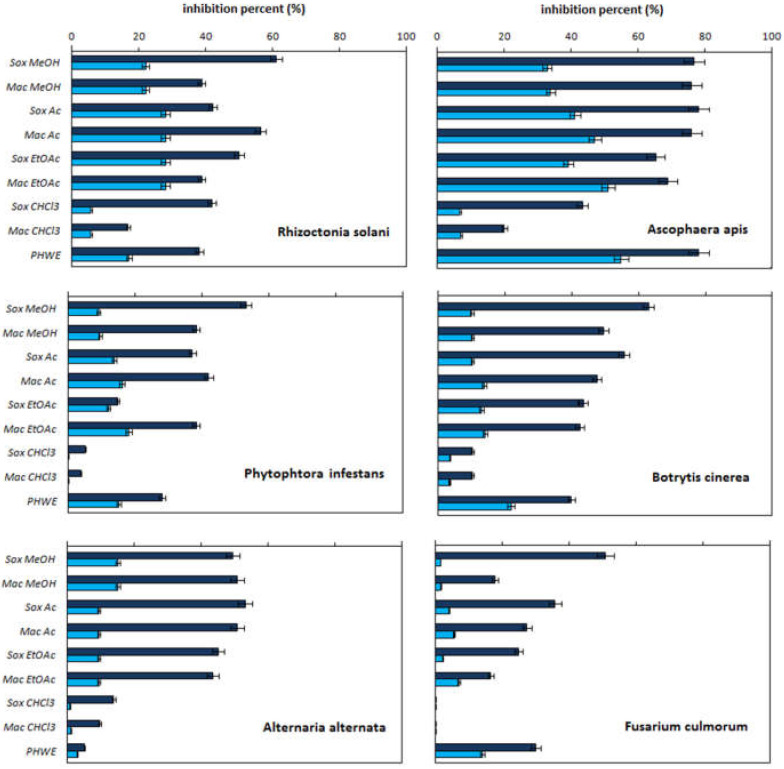
Comparison of the inhibition activity of mycelium growth by the white onion scale extracts (navy blue bars) obtained by pressurized hot water extraction (PHWE), extraction in the Soxhlet apparatus (Sox), and maceration (Mac) using different extractants [methanol (MeOH), acetone (Ac), ethyl acetate (EtOAc) or chloroform (CHCl3)] and by the quercetin solutions (blue bars) with the same quercetin concentrations as those in the onion scale extracts.

**Figure 2:**
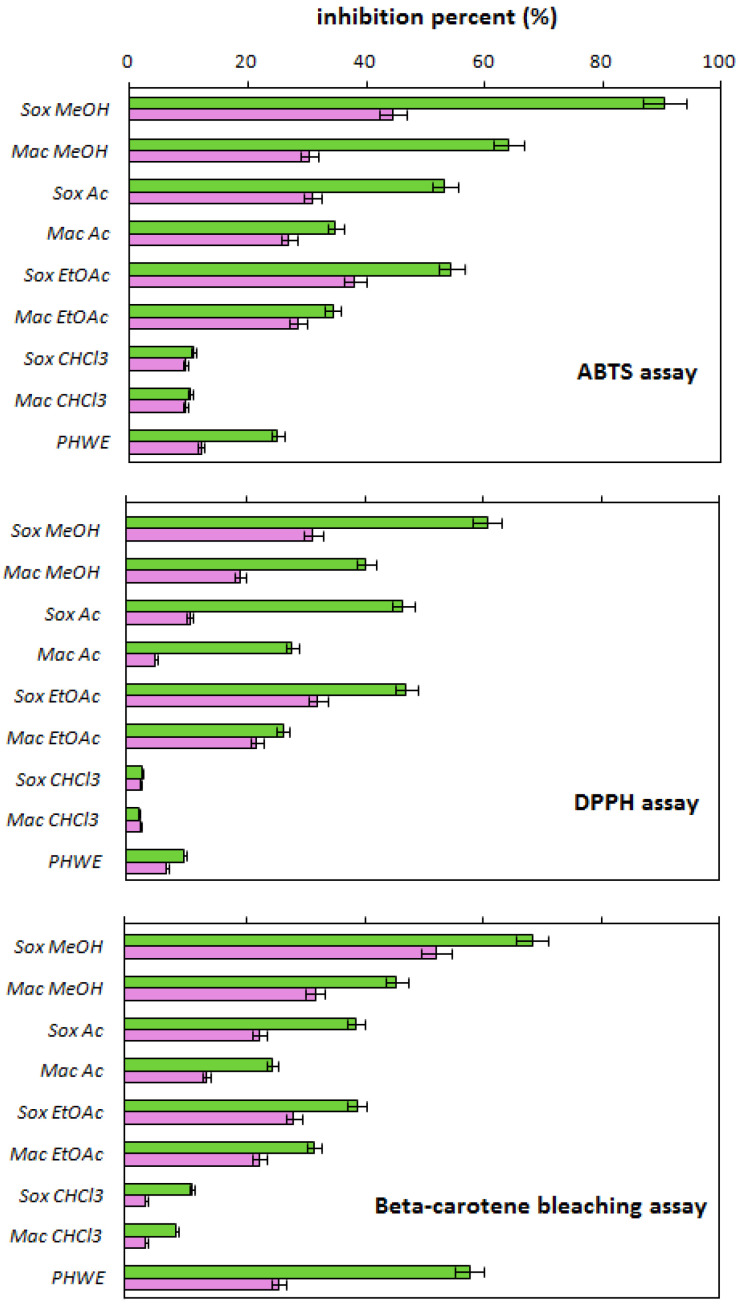
Comparison of the antioxidant activity of white onion scale extracts (green bars) obtained by the pressurized hot water extraction, extraction in the Soxhlet apparatus, and maceration using different extractants (methanol, acetone, ethyl acetate or chloroform) and by the quercetin solutions (pink bars) with the same quercetin concentrations as those in the onion scale extracts.

Figure 1 and Figure 2 summarize the antifungal and antioxidant activities of white onion scale extracts and quercetin solutions with the same quercetin concentrations as in the onion extracts, respectively (see column 4 in Table 1). The antioxidant activity was examined by the DPPH, ABTS, and beta-carotene methods. The antifungal activity was tested against various plant pathogenic fungi, such as *R. solani, B. cinerea*, *P. infestans*, *A. alternate*, and *F. culmorum*, as well as against the bee pathogenic fungi (*A. apis*). The results of these experiments are expressed as the inhibition percent (%). The statistical significance of the experimental factors determined according to the F value is summarized in Tables S1 and S2 for the antifungal and antioxidant activities, respectively. For the sake of clarity of discussion, the antifungal and antioxidant activities of the obtained extracts are discussed below in separate sections.

### 2.1. Antifungal Activity

The data presented in Figure 1 confirm the antifungal activity of the obtained extracts and the fact that this activity depends on the type of pathogen. In general, the highest susceptibility to the fungistatic action of the extracts is observed for *A. apis* (the highest bars), and the lowest for *F. culmorum*. The increase in the pathogen resistance, visible for *R. solani*, *P. infestans* and *A. alernata*, differentiates the fungistatic properties depending on the extraction technique and extractant. The results of the statistical analysis presented in Table S1 confirm this conclusion. The analysis shows that for each pathogen, the value of the Fisher’s coefficient, determined to assess the impact of the type of technique, exceeds the tabular (critical) value of this parameter.

The effectiveness of isolating compounds from a plant matrix depends on the extraction technique and the extractant used for this purpose. In general, the extraction efficiency increases with the temperature of the isolation process [12]. Meanwhile, in the light of the data collected in Figure 1, it appears that macerates are generally more active against most pathogens. Bearing in mind that the content of quercetin and phenols in macerates generally exceeds the content of these substances in extracts obtained in the Soxhlet apparatus (see Table 1), this fact can indirectly confirm the fungistatic activity of quercetin and phenolics.

Regardless of the isolation method, *A. apis* is the most sensitive pathogen to the change in the type of extractant (the highest *F_cal_* values in Table S1). It is worth noting here that even the water PHWE extract shows nearly 80% inhibition of the growth of this mycelium. For macerates, the pathogen most resistant to the solvent change is *F. culmorum* (the lowest *F_cal_* values in Table S1, see also Figure 1). In turn, the fungus most resistant to the change in the solvent used in the Soxhlet apparatus is *R. solani* (*F_cal_* = 29). The highest antifungal properties are exhibited by the MeOH extracts, and the weakest by the CHCl3 extracts. These differences result from their different selectivity for the isolation of active compounds. Nevertheless, the fact that a greater activity is observed for more polar liquids supports the key role of polyphenols, including quercetin, in the fungistatic activity of onion scale extracts. To test the fungistatic activity of quercetin alone, a series of solutions of the standard of this compound with concentrations corresponding to the amount of this compound in individual plant extracts (see Table 1) was prepared. This experimental approach facilitates the comparison of the inhibition activity of quercetin and the corresponding extract, and allows us to answer the question of whether quercetin is responsible for the activity of the mycelial growth inhibition by the examined extracts.

Comparing the results obtained for the onion extracts and the quercetin standard solutions, it can be concluded that, undoubtedly, quercetin exhibits fungistatic activity. However, the ability of quercetin solutions to inhibit mycelial growth is lower than that of onion scale extracts. Are there correlations between the quercetin content in the extracts and their fungistatic activity? The answer to this question is complex and difficult. On one hand, the fungistatic activity of the extracts correlates with the quercetin content in them (compare the quercetin content in the chloroform and methanol extracts with their activity). On the other hand, the quercetin solutions with distinctly different concentrations, corresponding to the content of this compound in the acetone and ethyl acetate extracts, exhibit similar activity against R. soloni (the differences are statistically insignificant). Therefore, there are no clear reasons for correlating the content of quercetin and polyphenols in the extracts with their biological activity in such a difficult research system, taking into account the interaction with a living organism. In view of the above, it can be postulated that other components of onion extracts, besides quercetin, exhibit mycelial growth inhibitory effects or that other components of onion extracts enhance the antifungal properties of quercetin. In other words, there is a synergistic antifungal effect of the remaining ingredients in the onion scale extracts. In the examined extracts, phenolics are strongly represented. These compounds are known for their antimicrobial properties [13]. It is possible that they also exhibit antifungal properties, inhibiting the mycelial growth or, they act synergistically with respect to the antifungal components.

### 2.2. Antioxidant Activity

The results of research on the antioxidant activity (AA) of white onion scale extracts obtained with various extraction techniques and solvents, including water, are shown in Figure 2. As can be seen, the change of the type of solvent and the extraction technique differentiates the properties of the extracts in a statistically significant way (see Table S2), and similarly to the previously presented results.

Comparing the activity of the extracts obtained in the Soxhlet apparatus to the activity of macerates, it can be seen, however, that regardless of the type of extractant, this time the extracts obtained at a higher temperature are characterized by a higher biological activity. Among all the tested methods, the highest activity (the highest values of the percent of inhibition, the highest bars) is shown by the beta(β)-carotene bleaching method, and the lowest by the DPPH method. This result is consistent with the previously published research results [10]. Regardless of the method of testing the antioxidant activity, the highest antioxidant properties are shown by the methanol extracts, and the weakest by the chloroform extracts. This conclusion, consistent with the previously presented results of studies on the fungistatic activity (FA) of the extracts, can constitute an indirect proof of the correlation of the amount of quercetin and polyphenols with the biological activity of white onion scale extracts. Nevertheless, when examining the antioxidant properties, it should be remembered that for a given method, the differences in AA, apart from the differences in the content of compounds in the extracts, are influenced by the solvent, which modifies the kinetics of the process of neutralization of reactive forms [14]. This effect was not observed in the antifungal studies, as the solvent of the extract was evaporated to dryness, and the dry residue was then dissolved in acetone inert to mycelium.

As it is known from the literature, the methods of antioxidant properties determination are based on two basic reaction mechanisms: single electron transfer (SET) and hydrogen atom transfer (HAT) [15]. The former is faster, the latter is slower. Both of these mechanisms can occur simultaneously, while the dominant mechanism in the examined system depends mainly on the used solvent, solubility, and partition coefficient. In general, the solvent with a high dielectric constant (ε) promotes the ionization/dissociation of the antioxidant, which is the main stage of radical neutralization in the ABTS and DPPH methods, which occurs mainly through the SET mechanism. Hence, it is not surprising that in these methods, high antioxidant properties are observed for the methanolic and acetonic extracts (ε = 32.6 and ε = 20.7, respectively) [16]. The chloroformic extracts exhibit the weakest antioxidant properties due to the fact that chloroform has a low extraction capacity and a low dielectric constant (ε = 4.8). In turn, in the β-carotene bleaching assay, the neutralization of peroxyl radicals (generated in the water emulsion) by the phenolic antioxidants occurs through the PCET (Proton Couple Electron Transfer) mechanism (a variant of the HAT mechanism). In this mechanism, the neutralization of the radical occurs through the formation of a complex between the radical and the antioxidant. Then hydrogen and electron are transported from the antioxidant to the radical after the homolytic breakdown of the bond [17]. Hydrogen transport is favoured in the solvent with the high β value, which expresses the acceptor capacity of hydrogen. Among the solvents used in studies, the low β parameter is characteristic of chloroform (β = 0.02) [18], while for methanol, acetone, and ethyl acetate the β values are 0.41, 0.5, and 0.45, respectively [19]. Hence, the better antioxidant properties are observed for the extracts obtained using these solvents.

In order to correlate experimentally the antioxidant activity of quercetin with that of white onion extracts, similarly to the previous series of studies, the activity of quercetin solutions with the concentrations corresponding to that of this compound in the individual onion extracts was tested. The antioxidant activity of quercetin is marked pink in Figure 2. The analysis of the presented results leads to the conclusion that, as before, the activity of quercetin is lower than that of the extracts. Nevertheless, the comparison of the ratio of the fungistatic activity of quercetin to the activity of extracts with the ratio of the antioxidant activity of quercetin to the activity of extracts reveals that in the latter case, the contribution of quercetin to the resultant antioxidant activity of the extracts is significant. This fact can be explained by the “simpler” test system based on chemical reactions rather than biological responses.

#### Correlation of Antioxidant Activity of Onion Extracts with the Temperature of PHWE Process and the Quercetin/Phenolics Content in the Extracts

The relation of the biological activity of the extract with the type of extractant and the technique of its preparation, presented above, makes it difficult to link unambiguously experimentally the content of quercetin and polyphenols in the extracts with their activity. This is confirmed, inter alia, by different biological activities observed for the aqueous extract obtained by the PHWE technique at 125 °C. This technique, unlike other tested extraction methods, allows control of the isolation efficiency of the same extraction medium by changing the extraction temperature. At this point, the question arises whether testing the antioxidant activity for a series of water extracts obtained at different temperatures will allow us to correlate their action with the content of the main and characteristic components in a better way. The results of this part of the research can be related to the process of thermal processing of vegetables during cooking; therefore, in addition to the inedible part of the onions, the study also included the edible part. Different varieties of onions differ in the content of compounds [20], therefore, for a broader insight into the correlations and antioxidant activity of onion extracts, the inedible and edible parts of the red onion were also studied.

**Figure 3:**
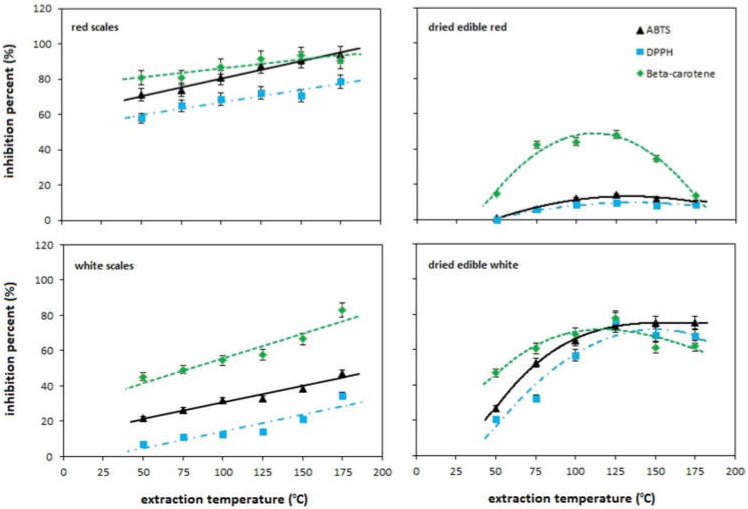
Effect of PHWE temperature on the antioxidant activities of white and red onion extracts determined using ABTS (black solid line with triangles), DPPH (blue dashed line with squares) and β-carotene bleaching assays (green dashed line with diamonds).

Figure 3 presents the antioxidant activities of onion extracts obtained for the inedible outer scales and dried edible interior of white and red onion bulbs at different temperatures of the PHWE process under the conditions of pressure, flow rate and extraction time: 100 Ba, 2.5 mL/min for 10 min. The antioxidant properties were determined using the DPPH, ABTS, and β-carotene assays, and expressed as the inhibition percent. Table 2 summarizes the amount of quercetin in the PHWE temperature extracts along with the total amount of phenols, both expressed per 1 g of biological matrix. The Pearson’s correlation coefficient was used to express the strength and direction of the linear relationship between AA and the quercetin/phenolics content. To assess how well these relationships can be described using a monotonic function, the Spearman’s rank correlation coefficient was used. The calculated values of the correlation coefficients are presented in Table 3.

Analyzing the relations in Figure 3, it can be seen that the antioxidant activity of the examined extracts depends on the method used to evaluate the activity, the extraction temperature, and the type of matrix. In all cases, the β-carotene bleaching assay reveals the highest antioxidant activity of the extracts (the highest value of inhibition percent). It appears that this can be related to the environment of the neutralization reaction itself, since the scavenging of the peroxide radical takes place in the water emulsion and the water serves as the extractant. For all extracts, the DPPH method shows the lowest activity. As far as the temperature effect is concerned, in the case of white and red onion scales, its increase is conducive to the increasing antioxidant properties in the entire studied temperature range. For the edible parts, the maximum properties are in the range of 75 °C to 125 °C for red onions and 100–150 °C for white onions. Above these temperature ranges, the antioxidant activity of the extracts decreases. As for the effect of the matrix type on the antioxidant activity, in the case of inedible parts of onions, red onion scale extracts have greater antioxidant properties. On the other hand, in the case of the edible parts, the relationship changes and this time the extracts of white onion show greater antioxidant properties. The observed changes in antioxidant properties are related to the change in the efficiency of extraction/degradation of compounds with the temperature of the process and differences in the composition of compounds characteristic of both onion varieties.

Comparing the trends in the changes in the antioxidant properties with the content of quercetin/polyphenols in the extracts (see Table 2), it seems that quercetin is responsible for the antioxidant activity of the extracts. The comparison of the values of the correlation coefficients collected in Table 3 proves unequivocally that not only quercetin but also polyphenols are responsible for the biological activity of onion extracts. In the vast majority of cases, observed very large positive correlations (at the significance level *p* < 0.05) were observed with both quercetin and the total content of polyphenols. In the case of quercetin, the values of the Pearson’s and Spearman’s coefficients complement each other, proving either linear or monotonic dependencies, while in the case of polyphenols, linear correlations dominate.

## 3. Materials and Methods

### 3.1. Plant Materials and Chemicals

Portions of white and red onion bulbs (*Allium cepa* L.) purchased at a local market were peeled to obtain the edible insides and inedible outer scales of the onion. The edible insides of both onion varieties were separately air-dried at a controlled temperature and humidity, and then they were ground. Inedible outer onion scales were ground and subjected to the extraction without drying. In both cases, sufficiently large sample portions were ground with a Braun cutting mill to the particle size of 0.2–0.4 mm. Thoroughly weighed portions of the samples were used for extractions.

Methanol, acetone, ethyl acetate, chloroform, 80% acetic acid, sodium carbonate, potassium persulfate (all of analytical reagent grade), and acetonitrile (HPLC grade) were purchased from the Polish Chemical Plant POCh (Gliwice, Poland). The Folin–Ciocalteu’s phenol reagent, linoleic acid, Tween 20, β-carotene, di-potassium peroxdisulfate, 2,2′-azinobis(3-ethylbenzothiazoline-6-sulfonic acid) diammonium salt (ABTS), and 2,2′-diphenylpicrylhydrazyl (DPPH) were purchased from Sigma Aldrich (Poznań, Poland). Sabouraud dextrose agar was supplied by Argenta, CM0139 Oxoid, (Poznan, Poland). Water was purified using a Milli-Q system from Millipore (Millipore, Bedford, MA, USA). Sand was obtained as a gift from glassworks (fraction 0.4–0.6 mm). The standard of quercetin was purchased from Merck (Darmstadt, Germany).

The plant pathogenic fungi [*Alternaria alternate* (Fries) Keissler, *Fusarium culmorum* (Smith) Saccardo, *Rhizoctonia solani* Kuhn, and *Botrytis cinerea* Persoon] were obtained from the Department of Phytopathology and Entomology, University of Warmia and Mazury in Olsztyn, Poland. *Phytophthora infestans* (anamorph) strain isolated from tomatoes was provided by the Main Inspectorate of Plant Health and Seed Inspection in Toruń (Poland). The fungus causing ascosphaeriosis in bees (*Ascosphaera apis* ATCC 13785) was supplied by the American Type Culture Collection (ATCC, USA).

### 3.2. Pressurized Hot Water Extraction

The PHWE system is described in detail in [10]. Extractions were performed in a dynamic mode on the weighed portions of edible and inedible parts of onion (0.5 g) which were mixed with sand and placed into the extraction vessels (22 mL). Each vessel was connected to the system and filled with water (1 mL/min). During pumping of water, the back pressure regulator was closed until the required pressure was reached in the system. Then the temperature was set to the desired level in the range 50–175 °C. To ensure the proper temperature inside the vessel, the set extraction temperature was 15 min equilibrated in the system (the equilibration time was optimized in separate experiments). After this time, the backpressure regulator was opened in such a way as to keep the pressure constant (40 bar). The extraction time (10 min) was counted from the opening of the backpressure regulator. The flow rate was 1 mL/min.

The collected extracts were evaporated to dryness using the rotary vacuum evaporator (Buchi R II rotavapor; 40 °C). Each extraction procedure was repeated three times using fresh portions of plant material.

### 3.3. Extraction in the Soxhlet Apparatus

Exhaustive extractions in the Soxhlet apparatus were performed using 70 g portions of the white onion scales, with either methanol, acetone, ethyl acetate, or chloroform as the extractant (200 mL). Each sample was extracted for 6 h. After cooling, the extract was removed and a new portion of the fresh extractant was added to the remaining material. The obtained extracts were pooled together and evaporated to dryness. For the statistical purposes, three independent extractions were performed.

### 3.4. Maceration

A 70 g portion of the white onion scales was immersed in either methanol, acetone, ethyl acetate, or chloroform (200 mL) for 24 h at room temperature. After this time the extract was removed, filtered through Whatman no. 4 paper, and evaporated to dryness. For the statistical purposes, three independent extractions were performed.

### 3.5. HPLC Analysis of Quercetin

HPLC measurements were performed with a Dionex liquid chromatograph DX600 (Dionex Corp., Sunnyvale, CA, USA) described in detail in [5,9]. The identification of the quercetin peak was made by comparing the retention time of the peak and its UV–Vis spectra with those of the quercetin standard. The retention time of quercetin was 10.7 min. The quercetin concentrations in the resulting extracts were calculated from the calibration curve, which was constructed using six standard solutions of quercetin in the concentration range 1.44–144 µg/mL.

### 3.6. Total Phenolics Content Measurement

The total polyphenol concentration in the extracts was determined using the Folin–Ciocalteu (F–C) method [21]. Briefly, a portion of 0.02 mL of extract was diluted with 1.58 mL of water and mixed with 0.1 mL of the F–C reagent. Then, 0.3 mL of 20% *w*/*v* sodium carbonate aqueous solution was added to the mixture. After incubation for 2 h at room temperature, the absorbance was measured at 765 nm using the spectrophotometer. Quantification was performed with respect to the standard curve of gallic acid (0.001–0.010 mg/mL). The results were expressed as gallic acid equivalents (GAEs), milligrams per gram of dry extract weight. The samples were measured in three replicates.

### 3.7. Antioxidant Activity

The antioxidant activity of white onion scale extracts as well as the quercetin solutions with the same quercetin concentrations as those in the onion extracts were assessed using the three spectrophotometric methods presented below. All measurements were performed in triplicate using the UV Probe-1800 Spectrophotometer (Shimadzu, Kyoto, Japan) and an optical glass cuvette (1 cm × 1 cm × 3.5 cm).

#### 3.7.1. β-Carotene Bleaching Assay

The stock solution of β-carotene/linoleic acid emulsion in water was prepared using: 25 µL of linoleic acid, 185 µL of Tween 20 (200 mg), and 5 mL of β-carotene solution (0.5 mg of β-carotene in 1 mL of chloroform) [17]. After evaporation of the chloroform, the residue was dispersed in 100 mL of distilled water which was saturated with oxygen (t = 30 min, F = 100 mL/min). The mixture was vigorously shaken, and 2900 µL of β-carotene/linoleic acid emulsion in water was mixed with 100 µL of extract. Then the whole mixture was placed in a cuvette, which was stoppered tightly and set in a water bath (45 °C). The decrease in β-carotene absorbance was monitored at 470 nm in the subsequent intervals (10 min) until the orange colour of the control sample disappeared (about 180 min). The control sample was prepared mixing 2900 µL of β-carotene emulsion with 100 µL of water. To zero the spectrophotometer, the emulsion without β-carotene but with water was prepared. The antioxidant activity was calculated as the inhibition percentage relative to the control.

#### 3.7.2. DPPH Method

2900 µL of DPPH^●^ methanolic solution (initial absorbance 0.7 ± 0.05 at 516 nm) was mixed with 100 µL of extract in a 4 mL test tube [22]. The whole was vigorously shaken for 30 s, then poured into a cuvette and placed in a spectrophotometer. The changes in DPPH^●^ were monitored at 516 nm in a continuous manner for 60 min with a spectrophotometer. Methanol (DPPH^●^ solvent) with water (or with another liquid used as an extractant) was used to zero the spectrophotometer.

#### 3.7.3. ABTS Method

The ABTS cation radical absorbance changes were monitored at 744 nm. The ABTS cation radical (ABTS^●+^) was prepared according to the procedure of Nenadis, et al. [23]. For this purpose, 5 mL of 7 mM aqueous ABTS solution was mixed with 88 μL of 140 mM potassium persulfate. After incubation for 16 h in the dark, and dilution with methanol to the absorbance value 0.7 ± 0.05 at 744 nm, the radical solution was prepared to be measured. During the measurements, 2900 µL of the methanolic solution of ABTS^●+^ was mixed in a 4 mL test tube with 100 µL of the extract. Pure methanol (ABTS^●+^ solvent) with water (or with another liquid used as an extractant) was used to zero the spectrophotometer. The percentage of ABTS^●+^ inhibition was calculated analogously to that of DPPH^●^.

### 3.8. Antifungal Activity

The activities of white onion scale extracts as well as the quercetin solutions against the plant and bees pathogenic fungi: *Botryti cinerea*, *Rhizoctonia solani*, *Alternaria alternata*, *Fusarium culmorum*, *Phytophthora cactorum* and *Ascosphaera apis* were tested in vitro according to the procedure described in [11,12]. The acetone solutions of dry extracts (with the concentration 10.0 mg/mL) and the quercetin standard (with the concentration from 0.06 to 10.0 mg/mL) were used in the study. A 1 mL aliquot of the solution was uniformly distributed on the surface of Sabouraud dextrose agar, and acetone was allowed to evaporate aseptically. The mycelial discs with a diameter of 5 mm were cut from a homogeneous culture of the fungus and placed on the agar medium surface, situated on sterile Petri plates with a diameter of 90 mm (Bionovo, Legnica Poland). The plates with only acetone deposited on the agar medium were used as a negative control. All plates were incubated at 25 °C for 5 days. The activity was determined by measuring the inhibitory zone diameter and was expressed as a percentage. The assay was performed in six replications.

### 3.9. Statistical Analysis

All experimental data are presented as the mean values of independent measurements ± standard deviation (SD). The diameters of the inhibition zones were measured using a ruler with an accuracy of 0.5 mm. The one-way analysis of variance (ANOVA) and the *F*-test were used to assess the influence of the experimental factors on the activity of extracts and the trend in the association between two ranked variables. If the calculated value of F (*F_cal_*) exceeds the table value F (*F_tab_*), this indicates a statistically significant influence of the given parameter. To determine the significance of each Fisher coefficient, the *p*-values were used. The values were considered to be significantly different when the result of the compared parameters differed at the *p* = 0.05 significance level. The Pearson’s and Spearman’s correlation coefficients were used to estimate the degree of association between the quercetin/phenolics concentration and the antioxidant activity of PHWE extracts obtained in the temperature range of 50–175 °C. The statistical analysis was performed using Excel (Microsoft Excel 2010).

## 4. Conclusions

In recent years there has been an increasing interest in diets rich in fruit and vegetables, which is mainly due to their role in the prevention of various degenerative diseases, such as cancer or cardiovascular disease. These health-promoting properties are associated with the presence of bioactive compounds, such as polyphenols, which translate into their use as functional food additives. Before using these compounds in specific applications, such as the food, pharmaceutical and cosmetic industries, they should be extracted from natural matrices and then analyzed and characterized. A particularly interesting phytochemical is quercetin, a powerful antioxidant, quite frequently found in many fruits and vegetables, including onions, which are one of the most commonly grown and consumed vegetables in the world.

In this study, various extraction techniques and various solvents classified as GRAS class reagents, including water, were used to extract quercetin from onions. It was shown that the fungistatic and antioxidant effects of onion scale extracts are related to the type of extractant and the technique of its preparation. The change in antioxidant properties of white and red onion extracts with the change in the temperature of the extraction process was proved, which can be related to the changes accompanying the process of thermal processing of vegetables and fruits for food purposes. Owing to the experimental and mathematical approaches to linking the main and characteristic components of onions with the biological activities of the extracts, it was possible to demonstrate their important effect concerning the antifungal and antioxidant properties. Considering that the research was carried out, inter alia, on the onion scales, showing a very high potential of biological properties of this waste material from agricultural production, the research results presented in this paper should encourage the use of this raw material, which has been so far underestimated by the industry for the production of various functional materials.

## Figures and Tables

**Table 1:** Concentration of quercetin and the total phenolics amounts in the examined extracts, expressed per 1 g of dry extract residue. The data are expressed as the mean values ± SD (*n* = 3).

Extractant Type	Extraction Method	Quercetin Content [mg/g]	Quercetin Concentration * [mg/mL]	Total Phenolics Content [mg GAEs/g]
CHCl3	Mac	3.05 ± 0.11	0.031 ± 0.001	57.2 ± 8.21
	Sox	2.56 ± 0.38	0.026 ± 0.004	57.6 ± 5.32
EtOAc	Mac	457 ± 3.75	4.570 ± 0.037	674 ± 9.60
	Sox	276 ± 0.71	2.760 ± 0.071	551 ± 9.23
MeOH	Mac	390 ± 1.98	3.90 ± 0.020	655 ± 8.12
	Sox	303 ± 1.27	3.03 ± 0.013	630 ± 2.82
Ac	Mac	147 ± 0.57	1.47 ± 0.006	414 ± 9.90
	Sox	139 ± 0.92	1.39 ± 0.009	486 ± 4.05
Water	PHWE	187 ± 1.16	1.87 ± 0.001	456 ± 5.48

* quercetin concentration in the solution obtained by dissolving 10 mg of dry extract in 1 mL of acetone (in the case of antifungal activity determination) or methanol or acetone or chloroform or ethyl acetate (in the case of antioxidant activity determination).

**Table 2:** Concentration of quercetin and the total phenolics amount in PHWE extracts obtained in the range of 50–175 °C from the edible and inedible parts of white and red onions.

Onion Variety	PHWE Temp. [°C]	Inedible Part		Edible Part	
		Quercetin Amount	Phenolics Amount	Quercetin Amount	Phenolics Amount
White onion	50	1.81 ± 0.05	19.8 ±0.21	3.22 ± 0.14	11.7 ± 0.22
	75	5.62 ±0.14	60.9 ± 3.15	3.53 ± 0.21	17.4 ± 0.55
	100	16.33 ±0.52	48.6 ± 2.85	3.63 ± 0.17	29.9 ± 1.24
	125	40.28 ±3.21	81.7 ± 3.47	3.61 ± 0.42	33.0 ± 1.23
	150	37.3 ± 2.98	83.6 ± 2.98	3.59 ± 0.27	23.0 ± 1.06
	175	28.5 ± 1.57	94.6 ± 3.46	3.48 ± 0.14	24.9 ± 0.98
Red onion	50	5.11 ± 0.13	61.7 ± 3.17	0.55 ± 0.09	2.34 ± 0.04
	75	9.97 ± 0.21	60.4 ± 2.85	2.35 ± 0.15	7.58 ± 0.05
	100	23.0 ± 1.54	75.6 ± 3.63	3.49 ± 0.22	28.7 ± 1.54
	125	31.0 ± 1.26	89.6 ± 3.47	3.57 ± 0.18	32.6 ± 0.95
	150	34.0 ± 0.98	97.2 ± 3.05	2.85 ± 0.13	18.3 ± 0.86
	175	30.0 ± 1.12	119 ± 0.26	2.43 ± 0.06	17.4 ± 0.44

**Table 3:** The Pearson’s (R) and Spearman’s (RHO) correlation coefficients (CCs) between the content of quercetin/phenolics and antioxidant activity determined by ABTS, DPPH, and β-carotene bleaching assays for the PHWE extracts obtained in the range of 50–175 °C from the edible and inedible parts of white and red onions (*p* < 0.05).

Onion Variety/Part	AA Method	CCs for Quercetin		CCs for Phenolics	
		R	RHO	R	RHO
White/inedible scales	ABTS	0.714 *	0.874	0.866	0.825
	DPPH	0.565 *	0.643	0.808	0.585
	Beta-carotene	0.641 *	0.837	0.827	0.655
White/edible entry	ABTS	0.842	0.071 *	0.820	0.270 *
	DPPH	0.816	0.110 *	0.875	0.532 *
	Beta-carotene	0.860	0.800	0.950	0.760
Red/inedible scales	ABTS	0.951	−0.217 *	0.961	−0.424 *
	DPPH	0.856	0.364 *	0.908	0.294 *
	Beta-carotene	0.990	0.517 *	0.833	0.650
Red/edible entry	ABTS	0.948	0.829	0.933	0.956
	DPPH	0.942	0.789	0.841	0.901
	Beta-carotene	0.758 *	0.669	0.610 *	0.524 *

* *p* > 0.05.

## Data Availability

Not applicable.

## Supplementary Materials

The following supporting information can be downloaded at: https://www.mdpi.com/article/10.3390/molecules27238164/s1. Table S1: F-values (*p*-values) obtained during the variance analysis of the influence of maceration and extraction in the Soxhlet apparatus, as well as that of the extraction solvent type: methanol, acetone, ethyl acetate, and chloroform, on the fungistatic activity (FA) of the onion scale extracts against various pathogenic fungi. Table S2: F-values (*p*-values) obtained during the variance analysis of the influence of maceration and extraction in the Soxhlet apparatus, as well as that of the extraction solvent type: methanol, acetone, ethyl acetate, and chloroform, on the antioxidant activity (AA) of onion scale extracts assessed by different methods (* *F_tab_* = 4; ** *F_tab_* = 8).

## Sample Availability

Samples of the compounds are not available from the authors.
