# Supplementary material for: Correlation Study of Biological Activity with Quercetin and Phenolics Content in Onion Extracts
Source: Molecules. 2022 Nov 23;27(23):8164. doi: 10.3390/molecules27238164 (PMC9739139; doi:10.3390/molecules27238164)
Supplement: Supplementary file 1 [file molecules-27-08164-s001.zip › molecules-2004643-supplementary.pdf]

Supplementary Materials:

Table S1. F-values (p-values) obtained during the variance analysis of the influence of maceration and extraction in the Soxhlet apparatus as well as that of the extraction solvent type: methanol, acetone, ethyl acetate and chloroform on the fungistatic activity (FA) of the onion scale extracts against various pathogenic fungi (*F_{tab} = 4; ** F_{tab} = 8).

**Effects**		**B. cinerea**	**F. culmorum**	**R. solani**	**A. alternata**	**P. infestans**	**A. apis**
* Solvent type on the FA of extracts obtained during:	Mac	319 ($1·10^{−08}$)	15 ($1·10^{−03}$)	287 ($2·10^{−08}$)	575 ($1·10^{−09}$)	133 ($4·10^{−07}$)	1463 ($3·10^{−11}$)
	Sox	926 ($2·10^{−10}$)	160 ($2·10^{−07}$)	29 ($1·10^{−04}$)	403 ($5·10^{−09}$)	120 ($6·10^{−07}$)	1164 ($7·10^{−11}$)
** Extraction technique on the FA of extracts obtained using:	MeOH	91 ($7·10^{−04}$)	660 ($1·10^{−05}$)	100 ($5·10^{−04}$)	2 ($3·10^{−01}$)	30 ($6·10^{−03}$)	4 ($1·10^{−01}$)
	Ac	33 ($5·10^{−03}$)	2 ($3·10^{−01}$)	29 ($6·10^{−03}$)	8 ($5·10^{−02}$)	16 ($2·10^{−02}$)	4 ($1·10^{−01}$)
	EtOAc	$4·10^{−01}$ ($6·10^{−01}$)	14 ($2·10^{−02}$)	40 ($3·10^{−03}$)	1 ($3·10^{−01}$)	36 ($4·10^{−03}$)	28 ($6·10^{−03}$)
	CHCl3	0 (1)	$7·10^{04}$ (1)	$9·10^{08}$ ($7·10^{−18}$)	9 ($4·10^{−02}$)	2 ($3·10^{−01}$)	1 ($5·10^{−06}$)

Table S2. F-values (p-values) obtained during the variance analysis of the influence of maceration and extraction in the Soxhlet apparatus as well as that of the extraction solvent type: methanol, acetone, ethyl acetate, and chloroform on the antioxidant activity (AA) of onion scale extracts assessed by different methods (*F_{tab} = 4; ** F_{tab} = 8)

**Effects**		**ABTS**	**DPPH**	**β-carotene**
* Solvent type on the AA of extracts obtained during:	Mac	350 ($8·10^{−09}$)	389 ($6·10^{−09}$)	298 ($2·10^{−08}$)
	Sox	362 ($7·10^{−09}$)	377 ($6·10^{−09}$)	330 ($1·10^{−08}$)
** Extraction technique on the AA of extracts obtained using:	MeOH	189 ($2·10^{−04}$)	93 ($6·10^{−04}$)	91 ($7·10^{−04}$)
	Ac	283 ($7·10^{−05}$)	142 ($3·10^{−04}$)	112 ($4·10^{−04}$)
	EtOAc	322 ($6·10^{−05}$)	176 ($2·10^{−04}$)	69 ($1·10^{−03}$)
	CHCl3	2 (0.27)	14 ($2·10^{−02}$)	40 ($3·10^{−03}$)
